# Tube pancreaticogastrostomy: A novel approach for laparoscopic duodenum-preserving total pancreatic head resection

**DOI:** 10.1097/MD.0000000000049087

**Published:** 2026-06-12

**Authors:** Zhongqiang Xing, Zixuan Hu, Xueqing Liu, Jianhua Liu

**Affiliations:** a Hepatobiliary Surgery Department, Second Hospital of Hebei Medical University, Shijiazhuang, Hebei, China.

**Keywords:** cystic neoplasms, duct-to-mucosa anastomosis, laparoscopic duodenum-preserving pancreatic head resection, neuroendocrine neoplasms, pancreaticogastrostomosis

## Abstract

Laparoscopic duodenum-preserving pancreatic head resection avoids complicated organ resection and reconstruction. In recent years, it has replaced laparoscopic pancreaticoduodenectomy as the first choice for treating benign and low-grade pancreatic head tumors. During the operation, pancreaticojejunostomy (PJ) is performed, and a small amount of pancreatic head is retained to ensure blood supply. In this study, we innovatively apply modified pancreaticogastrostomy (PG) and remove the pancreatic head further to reduce the occurrence of pancreatic fistula and intraoperative trauma. Clinical data of 53 patients who received laparoscopic duodenum-preserving total pancreatic head resection in our hospital from May 2020 to December 2023 were retrospectively analyzed, including sex, age, operation time, and postoperative complications. According to the reconstruction mode of the residual pancreatic duct, the patients were classified into a modified PG group and a PJ group. There were 25 cases in the modified PG group and 28 in the PJ group. The operation was completed in all patients, with no conversion to laparotomy. The anastomotic time of modified PG was significantly shorter than that of PJ (15.4 ± 0.8 min vs 35.1 ± 4.8 min, *P* < .05), and the postoperative hospital stay in the modified PG group was also significantly shorter than that in the PJ group (10.1 ± 2.2 min vs 15.1 ± 9.1 min, *P* < .05). There was no significant difference in the overall incidence of postoperative pancreatic fistula (POPF) between the 2 groups, the rates being 10.7% and 8.0% in the PJ and modified PG groups, respectively. One patient underwent reoperation due to abdominal hemorrhage caused by POPF in the PJ group, and another patient in the modified PG group underwent reoperation due to postoperative gastric fistula. The study provides preliminary evidence of the safety and effectiveness of the modified PG method, leading to shorter gastrointestinal reconstruction and postoperative hospitalization time. However, it is essential to note the limitations of the small sample size and short-term follow-up. Future studies should expand the sample size and assess the long-term outcomes of this anastomosis method.

## 1. Introduction

In recent years, laparoscopic duodenum-preserving pancreatic head resection (LDPPHR) has replaced laparoscopic pancreaticoduodenectomy (LPD) as the preferred surgical treatment for benign and low-grade malignant tumors of the pancreatic head.^[1]^ The traditional pancreaticojejunostomy (PJ) method has a certain risk of postoperative pancreatic fistula (POPF), which has not been fully resolved. It can lead to delayed gastric emptying, abdominal infection, bleeding, septic shock, prolonged hospital stay, increased medical costs, and even death, seriously impairing patients’ quality of life and survival.

To address this issue, we performed modified duct-to-mucosa pancreaticogastrostomy (PG) in LDPPHR, incorporating our previous experience with the use of drainage tubes in PJ.^[2]^ The PG method shortens the operation time and reduces the incidence of POPF. Although applying PG in pancreatic surgery is relatively rare, it has unique advantages over PJ. Some meta-analyses and randomized controlled trials have shown that the incidence of POPF in PJ is higher than in PG.^[3,4]^

## 2. Methods

### 2.1. Initial population

Clinical data of 53 patients who received laparoscopic duodenum-preserving total pancreatic head resection (LDPPHRt) in our hospital from May 2020 to December 2023 were retrospectively analyzed, including sex, age, operation time, and postoperative complications. According to the reconstruction mode of the residual pancreatic duct, the patients were divided into the modified PG group and the PJ group. There were 25 cases in the modified PG group and 28 in the PJ group.

The inclusion criteria were as follows:

1. The age range of patients was 18 to 75 years old.
2. Patients generally tolerated surgery.
3. Preoperative abdominal contrast-enhanced computed tomography or magnetic resonance imaging demonstrated benign or low-grade malignant tumors of the pancreatic head, such as solid pseudopapillary neoplasia, intraductal papillary mucinous neoplasia, and serous cystic adenoma.

The exclusion criteria were as follows: patients with chronic pancreatitis, considering that inflammation of the pancreatic head increases the difficulty of LDPPHRt; patients transitioning to laparotomy or LPD; and patients with abnormal cardiopulmonary function.

### 2.2. Surgical procedures

#### 2.2.1. Patient position and trocar layout

After successful anesthesia, the patient was placed in a high head and low foot position. The surgeon stood on the right side of the patient. The assistant and the scope assistant were positioned on the patient’s left side and between his legs. Five trocars were symmetrically distributed, with the observation trocar 1 cm below the umbilicus as the center. The operator’s and assistant’s right-hand operation trocars were all 12 mm, and the left-hand operation trocars were all 5 mm. A 3D lens was used during the operation.

#### 2.2.2. Preparation before pancreatic head resection

An ultrasound scalpel was used to open the gastrocolic ligament to the right fusion fascia, exposing the pancreatic head and neck. Without damaging the duodenal peritoneum, the posterior peritoneum of the lower margin of the pancreas was opened to expose the superior mesenteric vein and the Henle trunk. The anterior superior pancreaticoduodenal vein (ASPDV) and the right gastroepiploic vein were located along the surface of the pancreatic head, and the gastropancreatic trunk was ligated and cut off. The post-pancreatic neck tunnel was opened, and an ultrasonic scalpel severed the pancreatic parenchyma. The main pancreatic duct (MPD) was sharply severed with scissors.

#### 2.2.3. Pancreatic head resection

The direction of pancreatic head resection is from the bottom left to the top right – the separation of pancreatic tissue in the connective tissue between the duodenum and the pancreatic head. The MPD near the duodenal papilla was carefully identified and ligated, and the ampulla and common bile duct (CBD) were located along the MPD. Then, pancreatic tissue around the CBD and duodenum was excised. Suppose the CBD is located inside the pancreas. In that case, the CBD can be located behind the gastroduodenal artery and on the right side of the portal vein, combined with the proximal and distal directions to free the CBD. The gastroduodenal artery was found at the superior pancreatic margin, and the anterior superior pancreaticoduodenal artery was severed. The operation area was irrigated after specimen resection. The blood supply of the duodenum and CBD was observed, and whether there was a biliary fistula was carefully noted. After the specimen was removed, the pancreatic stump was sent for intraoperative frozen section. For those patients in whom malignant lesions were suspected or who had postoperative biliary or duodenal ischemia, the operation was switched to LPD. For patients with negative margins, pancreatic anastomosis was performed after the pancreatic stump was dissociated by approximately 3 cm.

#### 2.2.4. The modified pancreaticogastrostomy

**Figure 1.**
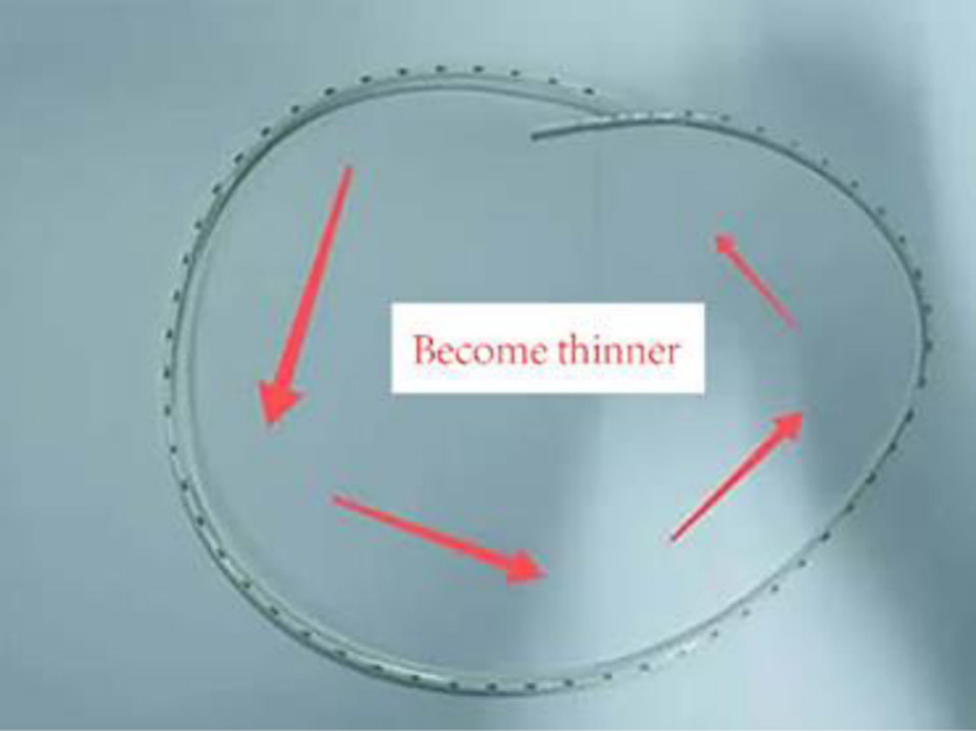
Pancreatic drainage tube used in pancreaticogastrostomy – variable diameter measurable pancreatic duct.

**Figure 2.**
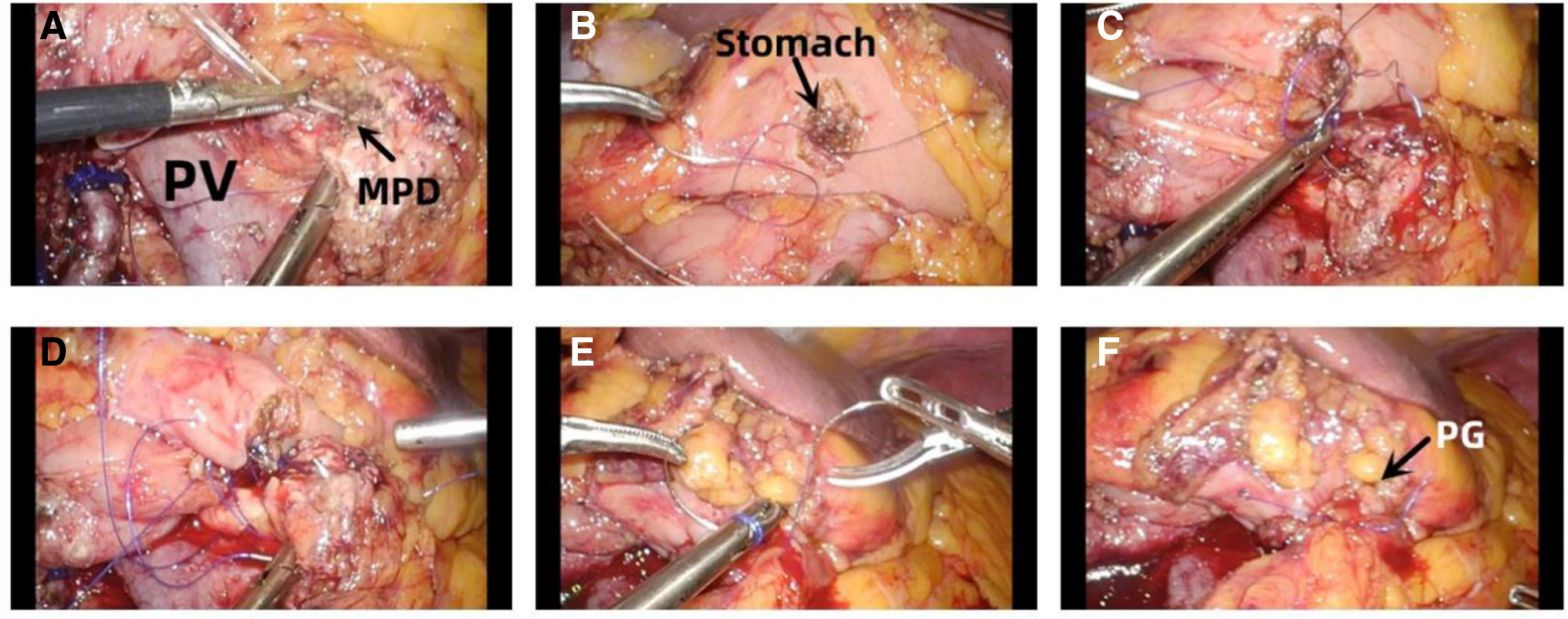
(A) The suture penetrated the whole layer of the pancreas and the pancreatic drainage tube. (B) Purse-string suturing was performed at the hole of the mucosa side of the posterior gastric wall without knotting. (C) Fixed ventral pancreas and posterior wall of the stomach. (D) Fixed the dorsal pancreas and posterior wall of the stomach. (E) At the lower edge of the anastomosis, the ends of 2 sutures were knotted and fixed. (F) PG was completed. MPD = main pancreatic duct, PG = pancreaticogastrostomy, PV = portal vein.

In the modified PG group, according to the diameter of the opening of the MPD and the scale marked on the wall of the pancreatic drainage tube (Fig. 1), the corresponding part of the tube was cut off and inserted 4 to 5 cm into the MPD. After that, a 4-0 absorbable suture was used to run through the whole layer of the pancreas and the pancreatic drainage tube and was knotted beside the tube for fixation (Fig. 2A). The corresponding position of the MPD opening was located on the posterior wall of the stomach, and a 4 cm^2^ gastric serous layer was removed. A hole was drilled in the area where the serous layer was removed. The diameter of the hole could accommodate the insertion of the pancreatic drainage tube. Then, purse-string suturing was performed around the hole with a 4-0 absorbable suture and no knotting (Fig. 2B).

The ventral pancreas and the posterior wall of the stomach were sutured continuously with a 3-0 absorbable suture first (Fig. 2C). Then, the lateral part of the pancreatic drainage tube, approximately 3 to 4 cm in length, was placed into the stomach. The purse-string suture on the posterior wall of the stomach was tightened and knotted. Then, another 3-0 absorbable suture was used continuously to fix the dorsal pancreas and the posterior wall of the stomach (Fig. 2D). Finally, the ends of the 2 absorbable sutures at the upper and lower edges of the anastomosis were knotted and fixed (Fig. 2E,F).

#### 2.2.5. The pancreaticojejunostomy

In the PJ group, Roux-en-Y anastomosis was performed between the pancreatic stump and jejunum. First, the pancreatic drainage tube (Fig. 1) was placed into the MPD and fixed with a 4-0 absorbable suture through the MPD. Then, the jejunum was severed 20 cm away from Treitz ligament, and the stump of the jejunum was lifted to perform duct-to-mucosa PJ. A small hole was made at the corresponding position of the MPD on the stump of the jejunum, and a 4-0 absorbable suture was used to suture in a purse-string manner with no knotting. The dorsal pancreas was fixed to the intestinal wall using a 3-0 absorbable suture, and then the pancreatic drainage tube was placed into the small intestine. The purse-string suture on the small intestine wall was tightened. Finally, another 3-0 absorbable suture was used to fix the ventral pancreas to the intestinal wall, and jejunal anastomosis was performed 50 cm from the PJ anastomosis and 20 cm from Treitz ligament.

After the operation, the abdominal cavity was irrigated. Whether there was wound bleeding or tissue ischemia was noted. Drainage tubes were routinely placed behind the anastomosis. The patients received routine treatments such as fluid replacement, anti-infection, parenteral nutrition, and somatostatin.

### 2.3. Follow-up and statistical analyses

In this study, follow-up involved outpatient reviews or telephone interviews. Contrast-enhanced computed tomography of the upper abdomen was performed 3 months after the operation. If necessary, electronic gastroscopy was used to evaluate the status of PG. SPSS26.0 was used to analyze the data in this study. The measurement data with a normal distribution were represented by ($\$\overset{―}{x}\pm s\$$), and the *t*-test was used for comparison between groups. The chi-square test was used for comparison of counting data between groups. The difference with a *P*-value < .05 was considered statistically significant.

## 3. Results

The 2 groups had no significant difference in preoperative characteristics (Table 1). The LDPPHRt was completed in all patients, and there was no conversion to laparotomy. The operation times in the modified PG and PJ groups were 300 (280, 320) min and 285 (250, 328.8) min, respectively, with no significant difference. The anastomotic time of modified PG was significantly shorter than that of PJ (15.4 ± 0.8 min vs 35.1 ± 4.8 min, *P* < .05) (Table 2). The postoperative hospital stay in the modified PG group was also significantly shorter than that in the PJ group (10.1 ± 2.2 min vs 15.1 ± 9.1 min, *P* < .05) (Table 3). There was no significant difference in the overall incidence of complications (21.4% vs 28.0%, *P* < .05), and the rates of POPF were 10.7% and 8.0% in the PJ and modified PG groups, respectively.

**Table 1:** Preoperative characteristics.

	Number	Male/female	Age (yr)	BMI (kg/m^2^)	History of abdominal surgery
Modified PG group	25	11/14	38.0 ± 11.7	21.9 (20.8, 24.0)	0
PJ group	28	9/19	34.5 ± 12.9	23.4 (21.4, 25.8)	1
*P* value		.273	.305	.215	.528

BMI = body mass index, PG = pancreaticogastrostomy, PJ = pancreaticojejunostomy.

**Table 2:** Intraoperative indicators.

Variable	Modified PG group	PJ group	*P* value
Operation time (min)	300 (280, 320)	285 (250, 328.8)	.368
Estimated bleeding (mL)	222.0 ± 122.5	258.9 ± 135.4	.305
Time of pancreatic anastomosis (min)	15.4 ± 0.8	35.1 ± 4.8	<.001
Diameter of MPD stump (mm)	3 (3, 4.5)	4 (3, 5)	.446
Conversion to laparotomy	0	0	Null
Intraoperative transfusion	0	0	Null

MPD = main pancreatic duct, PG = pancreaticogastrostomy, PJ = pancreaticojejunostomy.

**Table 3:** Postoperative characteristics.

Variable	Modified PG group	PJ group	*P* value
Overall complications (n, %)	7 (28.0)	6 (21.4)	.579
Biochemical leakage (n, %)	2 (8.0)	2 (7.1)	.650
Pancreatic fistula (n, %)	2 (8.0)	3 (10.7)	.555
Grade B	2 (8.0)	2 (7.1)	.650
Grade C	0	1 (3.6)	.528
Bile leakage (n, %)	0	1 (3.6)	.528
Time of intestinal function recovery (h)	72 (48, 72)	72 (48, 90)	.963
Gastric fistula (n, %)	1 (4.0)	0	.472
Reoperation (n, %)	1 (4.0)	1 (3.6)	.726
Postoperative hospital stay (d)	10.1 ± 2.2	15.1 ± 9.1	.009
Tumor size (cm)	4.1 ± 1.5	5.2 ± 2.5	.060
30 d-mortality (n, %)	0	0	Null

PG = pancreaticogastrostomy, PJ = pancreaticojejunostomy.

One patient underwent reoperation due to abdominal hemorrhage caused by POPF in the PJ group, and another patient in the modified PG group underwent laparoscopic debridement and drainage surgery due to postoperative gastric fistula. No patient died within 30 days after the operation. Postoperative pathological results were as follows: solid pseudopapillary neoplasia of the pancreas in 17 cases, intraductal papillary mucinous neoplasia of the pancreas in 14 cases, serous cystic adenoma of the pancreas in 12 cases, mucinous cystic neoplasia of the pancreas in 8 cases, and pancreatic neuroendocrine tumors in 2 cases.

## 4. Discussion

DPPHR was first proposed by Professor Beger for treating benign lesions of the pancreatic head.^[5]^ DPPHR preserves part of the pancreatic tissue in the medial margin of the duodenum and behind the CBD to ensure blood supply and is accompanied by PJ. However, residual pancreas and PJ are the leading causes of POPF. Takada et al found that if the integrity of the posterior pancreaticoduodenal arterial arch was preserved during the operation, the blood supply to the distal CBD and ampulla could be ensured.^[6]^ Some surgeons also reported on LDPPHRt.^[7,8]^ Therefore, to improve the completion quality of DPPHR and reduce surgical trauma, we removed the pancreatic head and applied modified duct-to-mucosa PG. The PG avoids jejunum dissection and anastomosis, thus reducing the risk of enterostomy fistula. It also reduces the time of gastrointestinal reconstruction and postoperative hospitalization.

At present, the primary pancreatic anastomosis is PJ. Thanks to the extensive development of LPD, various PJ methods have been developed. However, there is no way to prevent POPF. At present, pancreatic fistula is still the most common serious complication after LPD, with an incidence of 5 to 25%.^[9]^ According to previous experience with secondary operations in patients with POPF, the leading causes of pancreatic fistula after PJ are intestinal wall edema leading to suture incision, poor biological healing, and obstruction of the input loop. Secondary abdominal infection, bleeding, and other complications caused by POPF also significantly increase the perioperative risk of patients; thus, POPF-related complications are the leading cause of postoperative death in patients.^[10]^ Compared with PJ, PG has natural advantages, such as the adjacent anatomical position of the stomach and pancreas, thick gastric wall and rich blood supply, slight anastomotic tension, and direct observation of the anastomotic stoma under gastroscopy after the operation. These features effectively avoid the disadvantages of PJ. Some randomized controlled trials have shown that PG has a significant advantage over PJ in reducing the incidence of POPF, and there is no significant difference between PG and PJ in postoperative complication rates and perioperative mortality.^[4,11,12]^

The existing methods of PG are mainly classified into 2 types: invagination anastomosis and duct-to-mucosa anastomosis. The binding PG reported by Professor Peng results in high-quality anastomosis and the absence of sutures on the gastric mucosa surface by allowing the pancreatic stump to be placed in the gastric cavity and enabling binding of the inside and outside of the gastric wall.^[13]^ However, the anterior and posterior walls of the stomach needed to be cut during DPPHR for anastomosis, which was challenging to complete under laparoscopy. Their surgical team thought PG increased the postoperative gastrointestinal bleeding risk.^[14]^ It may be related to the corrosion of the pancreatic stump by gastric juice. However, a new type of duct-to-mucosa PJ reported by Professor Hong^[15]^ in 2017 requires only approximately 30 minutes. Compared with the traditional PJ anastomosis time of approximately 60 minutes, the pancreatic anastomosis time is significantly shortened, and the operation is simple, which is more suitable for laparoscopic surgery. The key to Hong PJ is introducing pancreatic juice into the digestive tract with a pancreatic drainage tube and promoting biological healing of the anastomosis based on this mechanical connection.

Inspired by this, our team proposed a new duct-to-mucosa PG anastomosis based on the previous experience of Hong PJ in LPD. The modified PG is based on using the pancreatic drainage tube to form a firmly healing channel between the MPD and the stomach without interference from gastric and pancreatic juice. It has 2 key points: placement and fixation of the pancreatic drainage tube to prevent the leakage of pancreatic juice and purse-string suturing around the drainage tube at the posterior gastric wall to avoid gastric juice extravasation. The former uses the “variable diameter measurable pancreatic duct” (Fig. 1) invented by Professor Liu.^[2]^ The diameter of the tube is thick to thin, and the scale is marked on the tube wall so that the depth of placement in the MPD can be observed. A suitable diameter of the pancreatic drainage tube is selected to ensure close fitting with the MPD. In addition, a 3-0 absorbable suture is used to fix the pancreatic drainage tube through the MPD.

This modified PG offered the following benefits for high-quality LDPPHRt completion: Only the pancreatic drainage tube entered the gastric cavity, and the gastric wall was isolated between the pancreatic stump and the gastric juice. This way, bleeding from the pancreatic parenchyma caused by gastric juice corrosion could be avoided; The diameter of the hole in the posterior gastric wall was similar to that of the MPD, and there was no severe abdominal infection, even if an anastomotic fistula occurred; The suture did not penetrate through the whole layer of the gastric wall. Even if anastomotic edema occurred, the suture would not cut through the whole layer of the gastric wall and lead to the notch of the posterior gastric wall; and The steps of jejunal dissection and anastomosis after PJ were eliminated, and the operation time and postoperative hospital stay were shortened.

In this study, there were no grade C fistula patients in the modified PG group, which had certain advantages compared with 1 case (3.6%) in the PJ group. However, there was no statistical significance. The time of pancreatic anastomosis in the modified PG group was significantly shorter than in the PJ group (15.4 ± 0.8 min vs 35.1 ± 4.8 min, *P* < .05). A 2020 report on LDPPHR compared Hong’s PJ with pancreatic head duct and body duct end-to-end anastomosis.^[16]^ Similar to the modified PG, the end-to-end anastomosis did not involve jejunal dissection and anastomosis, and only a pancreatic drainage tube was used for pancreatic duct reconstruction. The anastomosing time was 18.2 ± 5.1 minutes, significantly better than that of Hong PJ group (27.5 ± 8.3 min). In addition, the modified PG group benefited from having the pancreatic drainage tube introduce pancreatic juice into the stomach, and there were no special requirements regarding the texture of the pancreas. The diameter of the pancreatic drainage tube invented by Professor Liu^[2]^ ranges from thick to thin, which renders it suitable for different pancreatic duct diameters.

Nevertheless, challenges persist in implementing the modified PG. The conservative treatment of gastric fistula is not effective. In this study, a patient developed a postoperative gastric fistula because the purse-string at the gastric wall was inadequately sutured. Although fully drained, reoperation was still required. To reduce intervention in the anastomotic site, only the accumulation of fluid and necrotic tissue around the anastomotic site was cleared and rinsed. After placing a flushing tube, the patient recovered and was discharged from the hospital.

## 5. Conclusion

The study provides preliminary evidence of the safety and effectiveness of the modified PG method, leading to shorter gastrointestinal reconstruction and postoperative hospitalization time. However, it is essential to note the limitations of the small sample size and short-term follow-up. Future studies should expand the sample size and assess the long-term outcomes of this anastomosis method.

## Author contributions

**Conceptualization:** Xueqing Liu, Jianhua Liu.

**Formal analysis:** Xueqing Liu.

**Resources:** Zixuan Hu.

**Software:** Zixuan Hu.

**Supervision:** Jianhua Liu.

**Writing – original draft:** Zhongqiang Xing.

**Writing – review & editing:** Zhongqiang Xing.
